# *Leptin* deficiency in CD8^+^ T cells ameliorates non-segmental vitiligo by reducing interferon-γ and Granzyme B

**DOI:** 10.3389/fimmu.2023.1158883

**Published:** 2023-05-03

**Authors:** Meiyu Wu, Lu Wang, Haijing Wu, Ming Yang, Zhenghao He, Yiran Chen, Huiming Zhang

**Affiliations:** ^1^ Department of Dermatology, Hunan Key Laboratory of Medical Epigenomics, The Second Xiangya Hospital, Central South University, Changsha, Hunan, China; ^2^ Hospital for Skin Diseases, Institute of Dermatoloy, Chinese Academy of Medical Sciences & Peking Union Medical College, Nanjing, China

**Keywords:** *Leptin*, vitiligo, CD8^+^ T cells, autoimmune disease, lipid metabolism

## Abstract

**Background:**

Vitiligo is an autoimmune skin disease mainly mediated by CD8^+^ T cells, which affects about 0.1%-2% population of the world. *Leptin* plays a critical role in regulating the activation of CD8^+^ T cells. However, the effect of *Leptin* on vitiligo remains unclear.

**Objectives:**

To explore the effect of leptin on CD8^+^ T cells and its influence on vitiligo.

**Methods:**

RNA sequencing and Quantitative Real-time PCR (RT-qPCR) were used to explore the differentially expressed genes. Immunofluorescence staining was performed on skin lesions. Leptin in serum was detected by enzyme linked immunosorbent assay (ELISA). The peripheral blood mononuclear cells were detected by flow cytometry after leptin stimulation for 72 hours. A vitiligo model was established by monobenzone on *Leptin* KO mice.

**Results:**

557 differentially expressed genes were found, including 154 up-regulated and 403 down-regulated genes. Lipid metabolism pathways showed a close relationship to the pathogenesis of vitiligo, especially the PPAR signaling pathway. RT-qPCR (p = 0.013) and immunofluorescence staining (p = 0.0053) verified that *LEPR* expressed significantly higher in vitiligo. The serum leptin level of vitiligo patients was significantly lower than that of healthy controls (p = 0.0245). The interferon-γ subset of CD8^+^LEPR^+^ T cells from vitiligo patients was significantly higher (p = 0.0189). The protein level of interferon-γ was significantly increased after leptin stimulation *in vitro* (p = 0.0217). In mice, *Leptin* deficiency resulted in less severe hair depigmentation. *Leptin* deficiency also resulted in significantly lower expressed vitiligo-related genes, such as *Cxcl9* (p = 0.0497)*, Gzmb* (p < 0.001)*, Ifng* (p = 0.0159), and *Mx1* (p < 0.001) after modeling.

**Conclusion:**

*Leptin* could promote the progression of vitiligo by enhancing the cytotoxic function of CD8^+^ T cells. *Leptin* may become a new target for vitiligo treatment.

## 1 Introduction

Vitiligo is an autoimmune skin disease with acquired depigmentation. The melanocytes in the skin of the patients are progressively destroyed, and the characteristic skin depigmentation spots with distinct boundaries and different shapes will appear (1). Vitiligo has a prevalence of about 0.1% to 2% in the population and can develop at any age (2). The lesions may remain stable for many years or progress slowly, without self-healing, often resulting in a heavy mental and psychological burden. Vitiligo can be classified into two main types, segmental and non-segmental (3). Since segmental vitiligo is less associated with autoimmune diseases (4), this study focused on non-segmental vitiligo, which is the most common type of vitiligo. The autoantibodies of vitiligo can lead to the occurrence of autoimmune complications (5, 6). Patients with vitiligo may develop insulin resistance and lipid metabolism disorders (7, 8), implying an important association between vitiligo and lipid metabolism disorders (9–11). So far, the controversial relationship between immunity and lipid metabolism in patients with vitiligo remains to be explored (12).

CD8^+^ T cells play a crucial role in the pathogenesis of vitiligo. Gene expression analysis of vitiligo lesions showed that interferon-γ (IFN-γ) and IFN-γ-induced genes, including T-cell chemokine receptor *CXCR3* and its multiple ligands, such as *CXCL9* and *CXCL10* were significantly upregulated (13, 14). Perforin and Granzyme B expressed by CD8^+^ T cells can lyse melanocytes directly (15). Secreted cytokines such as IFN-γ can recruit and activate Natural killer (NK) cells and Dendritic cells (DCs), and make vascular cell adhesion molecule 1 (*VCAM1*) expression higher, which in turn attracts more circulating memory CD8^+^ T cells and B cells to migrate to the lesion and strengthen the ability to remove melanocytes finally (16). These studies indicate that CD8^+^ T cells and their functional cytokines play an important role in the pathogenesis of vitiligo. However, the mechanisms of how these cytokines regulate CD8^+^ T cell activation and function are not fully understood.

Leptin is mainly synthesized and secreted by white adipose tissue, produced in proportion to white adipose tissue mass. Leptin is critical for metabolic homeostasis and can affect the differentiation and function of T cells (17, 18). Mattioli B et al. found that *Leptin* (*LEP*) could enhance the activation ability of CD8^+^ T cells (19). The proliferation and function of CD8^+^ T cells in mice and spleens and intestinal cavities of rats could be increased by *LEP* (20–22). *LEP* could enhance the function of CD8^+^ T cells by increasing the expression level of IFN-γ (23, 24), implying a notable relationship with autoimmune diseases. *LEP* may get involved in the pathogenesis of systemic lupus erythematosus by promoting T helper cell 17 (Th17) response and inhibiting Regulatory T (Treg) cells (25, 26). Studies also found that the serum leptin level in psoriasis patients was higher than that in healthy controls (27). Leptin may be a marker of the severity of psoriasis, and may also be a pathogenic cofactor leading to chronic psoriasis (28). Serum leptin levels in patients with alopecia areata were significantly higher than those in healthy controls (29). Serum leptin in patients with autoimmune thyroid disease was also significantly higher than those in patients without autoimmune thyroid disease (30). However, there are few studies on the association between leptin and vitiligo. Federica et al. suggested that vitiligo doesn’t seem to be associated with high body mass index (BMI), and elevated serum leptin levels don’t seem to be characteristic of vitiligo, in contrast to autoimmune diseases that are significantly associated with obesity (31). The effect of *Leptin* on the occurrence and development of vitiligo remains obscure.

This study attempted to investigate the expression of *Leptin*-related genes in patients with vitiligo, and further explored the regulatory effect of *Leptin* on CD8^+^ T cells and its influence on the occurrence and development of vitiligo. This study tried to find a new therapy for vitiligo by regulating systemic inflammation and oxidative stress through the lipid metabolism pathway.

## 2 Materials and methods

### 2.1 Vitiligo patients and healthy blood donors

The patients recruited in this study were non-segmental vitiligo. All the subjects were from the Second Xiangya Hospital and had no infectious diseases, metabolic secretory diseases, or other autoimmune diseases. The baseline characteristics of the participants are shown in **Table 1**. There was no significant difference in age, gender or BMI between all of the vitiligo patients and control groups (p > 0.05). Detailed information of each participant is provided in **Tables S1-5**. Informed consent was obtained from patients with vitiligo and healthy volunteers. The ethics permit for humans and mice was obtained from the Institutional Committee of Ethics of the Second Xiangya Hospital.

**Table 1:** Sample sizes and demographic characteristics of the participants.

	Controls	Vitiligo patients
Skin RNA sequencing (number)	3	3
Average age (years, mean age ± SEM)	27.33 ± 3.480	25.67 ± 2.186
Gender (males/females)	0:3	1:2
BMI (kg/m^2^)	19.13 ± 0.3724	19.71 ± 0.6450
Skin RT-qPCR (number)	9	12
Average age (years, mean age ± SEM)	24.17 ± 3.000	24.78 ± 3.161
Gender (males/females)	5:4	5:7
BMI (kg/m^2^)	20.90 ± 0.4920	19.98 ± 0.3120
Immunohistochemical staining (number)	5	5
Average age (years, mean age ± SEM)	35.40 ± 7.359	41.60 ± 8.846
Gender (males/females)	2:3	2:3
BMI (kg/m^2^)	19.96 ± 0.3134	19.84 ± 0.2097
Serum Leptin ELISA (number)	36	53
Average age (years, mean age ± SEM)	36.75 ± 1.186	33.38 ± 1.675
Gender (males/females)	17:19	25:28
BMI (kg/m^2^)	21.21 ± 0.2455	21.39 ± 0.1909

### 2.2 Mice and treatments

Six-week-old female C57 BL/6 WT black mice (N = 5) and *Lep* KO black mice of the same age and gender (N = 5) were used. The mice were purchased from Henan SCBS Biotechnology Co., Ltd., and kept in the SPF animal feeding barrier of the Second Xiangya Hospital. All animal experimental procedures were approved by the Ethics Committee of Xiangya School of Medicine. After 8 weeks of Monobenzone treatment, the mouse model of vitiligo was established, and the administration of Monobenzone was stopped for 1 week. In the 9th week of modeling, the spleens, the skin of hair depigmentation areas, and the marginal skin were extracted.

### 2.3 RNA isolation and RT-qPCR

The skin tissues of vitiligo patients were obtained from the dermatology biopsy room, while the normal skin tissues were from plastic and cosmetic surgeries. Under sterile conditions, the skin tissues were carefully peeled off with forceps, and excess fat was removed before being placed into cryotubes, stored in a -80°C liquid nitrogen for RNA isolation. The peripheral blood mononuclear cells (PBMCs) were from the clinical laboratory of the Second Xiangya Hospital and were separated using lymphocyte separation medium, Ficoll-Paque (GE Healthcare, US), and density gradient centrifugation. RNA was extracted using TRIzol Reagent (Meridian Life Science, US). RNA quality and quantity were detected by a NanoDrop ND-2000 spectrophotometer. Real-Time Quantitative PCR (RT-qPCR) was measured using SYBR Premix Ex TaqII (TaKaRa). The primers (TSINGKE) used were listed in **Table S6**. The Ct (cq) value of each reaction tube could be automatically calculated by Bio-rad real-time PCR instrument, and the relative gene expression was calculated according to 2^-ΔΔCt^ method.

### 2.4 Leptin treatment *in vitro*

Each tube of PBMCs from healthy donors was diluted with 20mL of sterile RPMI-1640 complete medium (Gibco, US) and 2mL of PBMCs was added in each well. The control group was treated with 1mL 4μg/mL phytohemagglutinin (PHA) solution and 1mL sterile RPMI-1640 complete medium. The experimental group was treated with 1mL 4μg/mL PHA solution and 1mL 0.91nM/mL leptin solution. There were about 10^6^ PBMCs in 1mL of RPMI-1640 complete medium. The samples were then cultured in a sterile cell incubator for 72 hours. After centrifugation, the supernatant was collected for ELISA assay.

### 2.5 Enzyme linked immunosorbent assay (ELISA)

Cytokines from PBMCs in culture supernatants were evaluated by ELISA quantification kits after being stimulated by leptin *in vitro* for 72 hours. Samples were diluted according to the instructions. The ELISA assays were performed with 3 replicates for each sample, which were averaged for comparison. Human Leptin ELISA Kit (CHE0053-096, Beijing 4A Biotech Co., Ltd.), Human Perforin-1 ELISA Kit (PRF1/PFP, Wuhan Huamei Biological Engineering Co., Ltd.), Human Granzyme B ELISA Kit (Beijing 4A Biotech Co., Ltd.), and Human IFN-γ ELISA Kit (Beijing 4A Biotech Co., Ltd.) were used.

### 2.6 Histology and immunofluorescence staining

Skin tissues from vitiligo patients or healthy controls were first treated with formalin and paraffin for hematoxylin and eosin staining (H&E) or immunofluorescence staining. Sections were then dewaxed with Turpentine oil, 95% ethanol, 75% ethanol, and 50% ethanol. Antigen thermal remediation was performed. Primary and secondary antibodies were then sequentially applied at room temperature (60 min). Recombinant Anti-Leptin Antibody (Beijing Yiqiao Shenzhou Technology Co. Ltd) and hLeptin R Mab (C1 52, R&D Systems, Inc.) were used. Slides were analyzed by a digital fluorescence microscope (Leica) and NLS Elements Basic Research Imaging Software (Leica).

### 2.7 Flow cytometry and confocal microscopy

After treated with surface antibodies at 4°C for 30 minutes, cells were prepared with Transcription Factor Buffer Set (BD Biosciences) according to the manufacturer’s instructions for intracellular staining, and then incubated with intra-nuclear antibodies for 45 min. Cells were collected with a Cytek Athena (Cytek Biosciences). FlowJo software (Tree Star) were used to analyze the flow cytometry results. The blank control (cells unstained), single-stained control (PC5.5, APC-CY7, PE-cy7-A, APC-A, PE and FITC), and Fluorescence-Minus-One (FMO) control were set. The panel was designed as PC5.5 (CD8), APC-CY7 (CD4), APC-A (LEPR second antibody), PE (perforin), FITC (Granzyme B), and PE-cy7 (IFN-γ). The following antibodies were used: Alexa Flour 647 Donkey anti-rabbit IgG (BioLegend), Rabbit Anti-Leptin receptor antibody (bs-0410R, Bioss), APC-Cy7 anti-human CD4 (BD Biosciences), Percp-Cy5.5 anti-human CD8 (BD Biosciences), PE anti-human Perforin (BD Biosciences), FITC anti-human/mouse Granzyme B (Granzyme B, BioLegend), PE-Cy7 anti-human IFN-γ (BioLegend), Zombie Aqua Fixable Viability Kit (BioLegend), APC-Cy7 anti-mouse CD4 (Abcam), Percp-Cy5.5 anti-mouse CD8 (Abcam), PE anti-mouse Perforin (Abcam), FITC anti-mouse/human granzyme B (BioLegend), PE-Cy7 anti-mouse IFN-γ (Abcam).

### 2.8 Isolation of mouse spleen CD8^+^ T Cells

Magnet-Activated Cell Sorting (MACS) were used to isolate the CD8^+^ T cells. CD8^+^ T cells were isolated from spleen samples of *Lep* KO and WT mouses using MACSxpress^®^ whole blood CD8 T cell isolation kit mouse (Miltenyi Biotec, Auburn, CA). All the non-CD8 T cells were immunomagnetically depleted with MACSxpress beads. To improve the purity, each sample was filtered through LS Separation columns more than three times. According to the instructions of the MACS kit, the purity rate can be more than 90%. Therefore, the purity of CD8^+^ T cells was not investigated again. The isolated CD8^+^ T cells were processed for RNA sequencing and RT-qPCR.

### 2.9 RNA sequencing and bioinformatics analysis

Three patients with vitiligo and three normal controls were recruited for RNA sequencing. Human skin and CD8^+^ T cells of mice were collected for RNA extraction, sample detection, enrichment, amplification, library preparation, and Illumina sequencing by Wuhan Huada Gene Technology Co. Ltd (Wuhan, China). All RNA seq data were analyzed by R Studio. To identify differentially expressed genes, the R package limma and edger were used. The package cluster Profiler was used for Kyoto Encyclopedia of Genes and Genomes and Gene Ontology enrichment analysis. P values < 0.05 and fold-change values >1.87 were used in the analyses.

### 2.10 Statistical methods

The data were presented as means ± SEM, unpaired t-test was used for two independent samples with normal distribution and homogeneity of variance. Paired t-test was used for two paired samples conforming to a normal distribution and homogeneity of variance. The Mann-Whitney U test was used for two independent samples that did not conform to a normal distribution and had unequal variances. Plots and statistical values were processed and represented by SPSS 25.0 (IBM Corp, Armonk, NY, USA). P values < 0.05 were considered statistically significant.

## 3 Results

### 3.1 Differential genes were enriched in lipid metabolism, especially the PPAR signaling pathway

**Figure 1:**
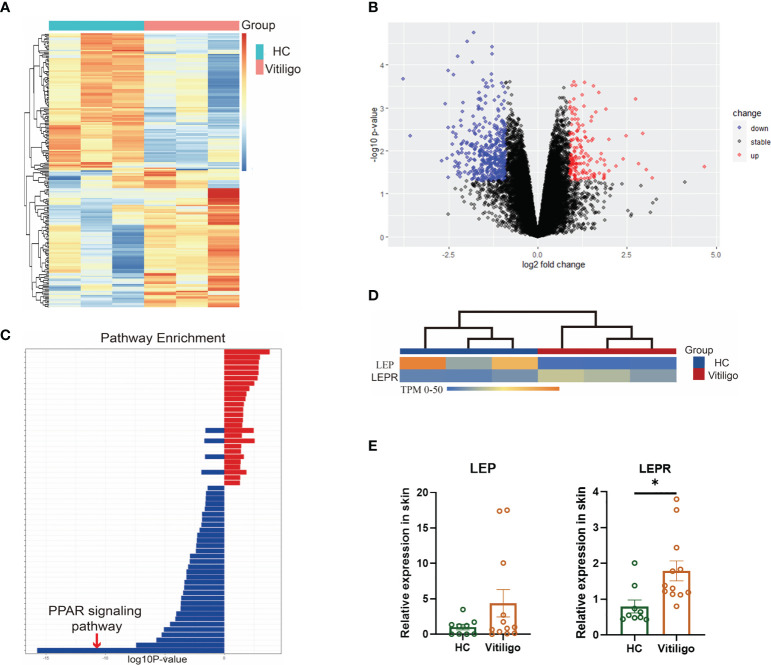
Differential genes were enriched in lipid metabolism, especially the PPAR signaling pathway. **(A)** Heatmaps of mRNA-seq analysis are shown, and significant differential clustering can be found between gene expression in patients with vitiligo and normal controls (Vitiligo: N=3, HC: N=3). **(B)** Volcano plots of upregulated (red) and downregulated (blue) genes are shown. **(C)** In the enrichment analysis of signal pathways, the top enrichment pathway was the PPAR signaling pathway. **(D)** There is a clear clustering of the expression of *LEP* and *LEPR* between vitiligo and healthy controls. **(E)** The different expression of *LEP* and *LEPR* were verified by RT-qPCR (Vitiligo: N=12, HC: N=9). ^*^P < 0.05.

The skin tissues from three vitiligo patients and three normal controls were used for RNA sequencing. A total of 557 differentially expressed genes were identified, including 154 up-regulated and 403 down-regulated genes (**Figures 1A, B**). Enrichment analysis showed that the differentially expressed genes in vitiligo groups were mainly enriched in peroxisomes, microbodies, and lipid droplets at the cell component level. In the biological process, it was mainly enriched in the lipid metabolic and small molecule catabolic process. At the molecular function level, it was mainly enriched in the activities of acyltransferases (except aminoacyl) and coenzyme binding. In the enrichment analysis of signal pathways, the top five enrichment pathways were: the PPAR signaling pathway, peroxisome, unsaturated fatty acid biosynthesis, fatty acid metabolism, and glycerolipid metabolism (**Figure 1C**). In the subsequent protein interaction analysis, the key differentially expressed genes in the interaction network were still enriched in fatty acid metabolism, lipid metabolism, PPAR signaling pathway, and regulation of lipid metabolism. Therefore, lipid metabolism is inextricably linked to the development of vitiligo, especially the PPAR signaling pathway. RT-qPCR results verified that *LEPR* was significantly highly expressed in total RNA derived from vitiligo skin lesions while *LEP* didn’t (**Figures 1D, E**, **Table S7**).

### 3.2 Increased *LEPR* expression in CD8^+^ T cells from vitiligo patients

**Figure 2:**
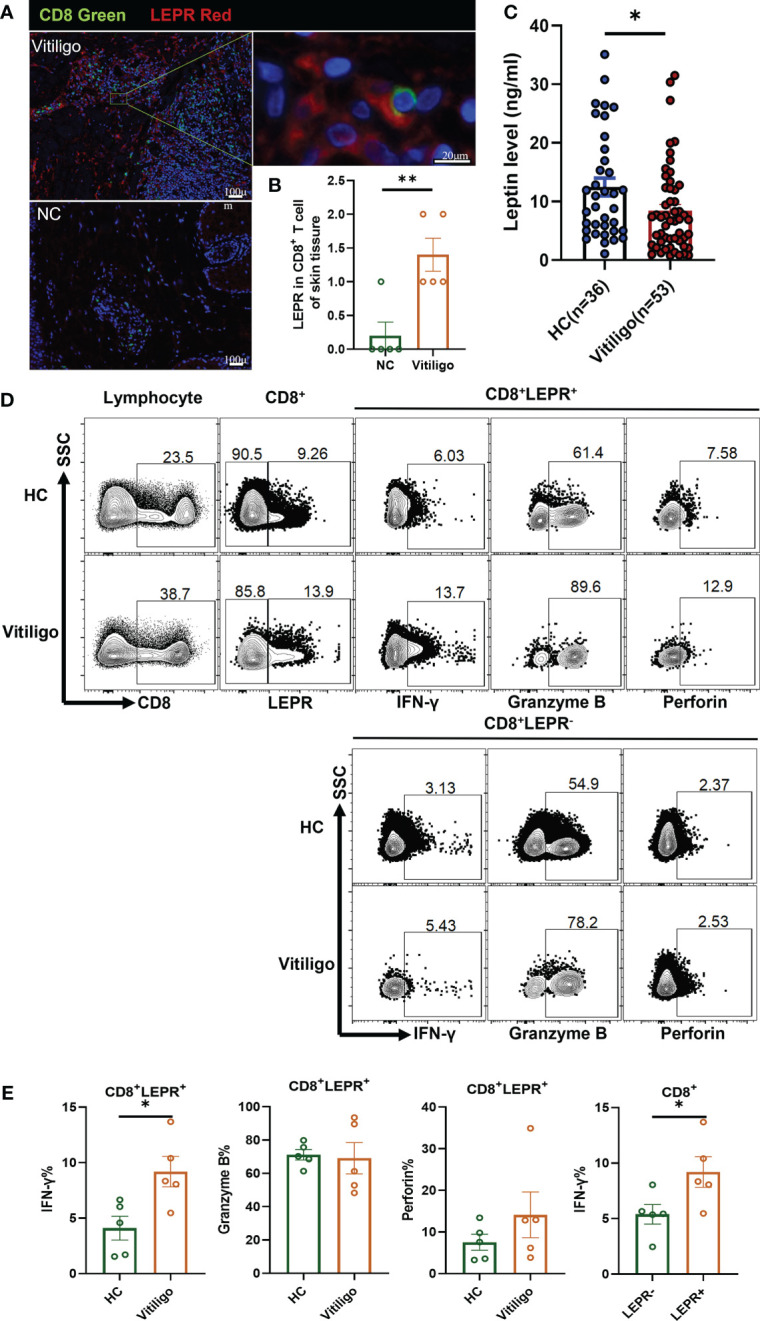
*LEPR* expression in CD8^+^ T cells from vitiligo patients was increased. **(A, B)** Double immunofluorescence staining results of *LEPR* and *CD8* in vitiligo lesions and normal skin (Vitiligo: N = 5, NC: N = 5). **(A)** *CD8* was represented by green fluorescence and *LEPR* was represented by red fluorescence. **(B)** The expression of *LEPR* in CD8^+^ T cells in Vitiligo lesions was significantly higher. **(C)** The serum levels of leptin were evaluated by ELISA (Vitiligo: N=53, HC: N=36). **(D, E)** Representative flow cytometric profiles and data plots of CD8^+^ T cells in PBMCs are shown (Vitiligo: N=5, HC: N=5). ^*^P < 0.05, ^**^P < 0.01.

As a free cytokine, the localization of *LEP* in skin tissue is difficult to be clearly shown in immunofluorescence staining. As an alternative, *LEPR* was stained, and CD8^+^ T cells were confirmed to express *LEPR* by double immunofluorescence staining of *LEPR* and *CD8*. *LEPR* in skin tissues of patients with vitiligo was significantly higher than that of healthy controls. (p = 0.0053, **Figures 2A, B**). However, serum leptin in vitiligo patients was significantly lower than in healthy controls (p = 0.0245, **Figure 2C**).

Flow cytometry showed that the *LEPR* expression in CD8^+^ T cells of PBMCs from patients with vitiligo was higher than that in healthy controls (**Figure 2D**). The frequency of IFN-γ in CD8^+^LEPR^+^ T cells of vitiligo patients was significantly higher than that in healthy controls (p = 0.0189, **Figure 2E**). The frequency of perforin in CD8^+^LEPR^+^ T cells was also higher than that in CD8^+^LEPR^-^ T cells. In addition, the frequency of IFN-γ in CD8^+^LEPR^+^ T cells was significantly higher than that in CD8^+^ LEPR^-^ T cells (p = 0.0480). More detailed information about the flow cytometry results is shown in **Table S8**. Compared with normal controls, CD8^+^LEPR^+^ T cells in the peripheral blood of patients with vitiligo showed enhanced expression of cytotoxic cytokines, and the expression of IFN-γ, Granzyme B, and perforin in CD8^+^LEPR^+^ T cells was higher than that in CD8^+^ LEPR^-^ T cells. *LEPR* probably promoted the secretion of cytotoxic cytokines from CD8^+^ T cells in patients with vitiligo.

### 3.3 Leptin enhances the expression of cytotoxic cytokines from CD8^+^ T cells *in vitro*

**Figure 3:**
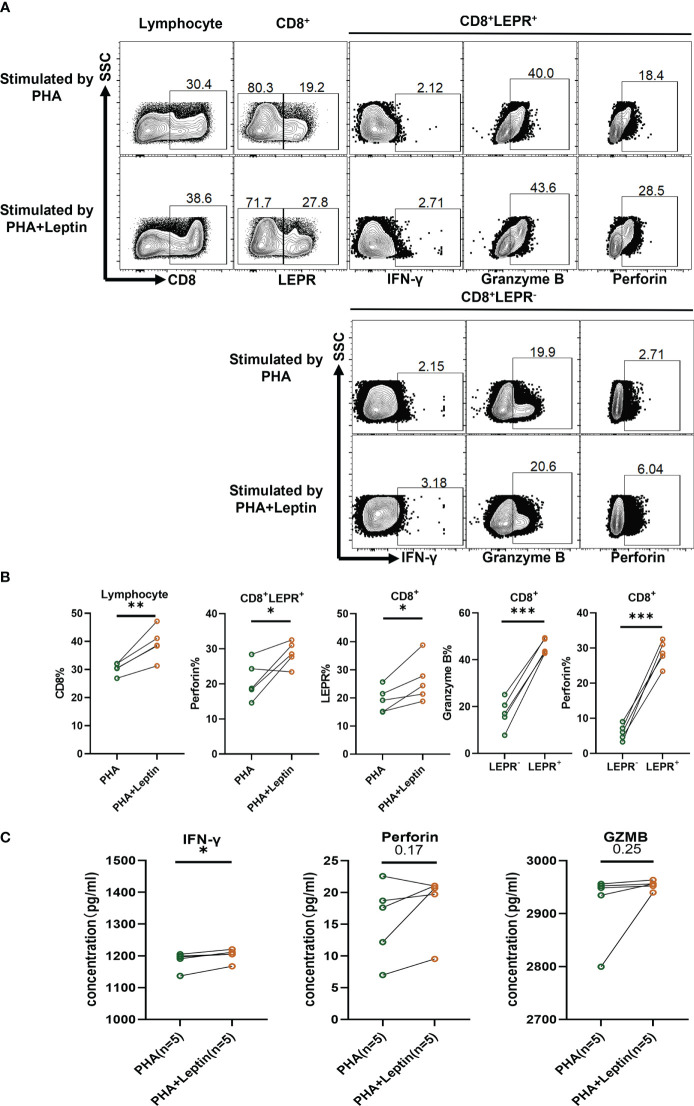
Leptin enhances the expression of cytotoxic cytokines from CD8^+^ T cells *in vitro*. **(A, B)** Gating strategy, representative flow cytometric plots, and statistical analysis of the percentage of cell subsets were shown. **(C)** After 72 hours of leptin stimulation, the protein levels of IFN-γ, perforin, and Granzyme B from normal PBMCs were detected by ELISA. ^*^P < 0.05, ^**^P < 0.01, ^***^P < 0.001.

The levels of perforin, Granzyme B, and IFN-γ secreted by PBMCs were evaluated after stimulation with leptin for 72 hours *in vitro*. The results of flow cytometry showed that the frequency of CD8^+^ T cells in the lymphocytes of the experimental group was significantly higher than that of the control group (p = 0.0070, **Figures 3A, B**). Leptin could promote the proliferation of CD8^+^ T cells. The frequency of *LEPR* from CD8^+^ T cells was significantly increased (p = 0.0248), indicating that the expression of *LEPR* had positive feedback on leptin stimulation. Leptin can also up-regulate the frequency of IFN-γ and perforin (p = 0.0444) in CD8^+^ T cells after stimulation. The detailed flow cytometry results are shown in **Table S9**. The protein levels of perforin, Granzyme B, and IFN-γ (p= 0.0217) in the experimental group were also higher than those in the control group according to ELISA assay (**Figure 3C**). In conclusion, the expression of CD8^+^ T cell functional molecules was significantly increased after leptin stimulation.

### 3.4 *Lep* deficiency in CD8^+^ T cells ameliorated vitiligo development in mice

**Figure 4:**
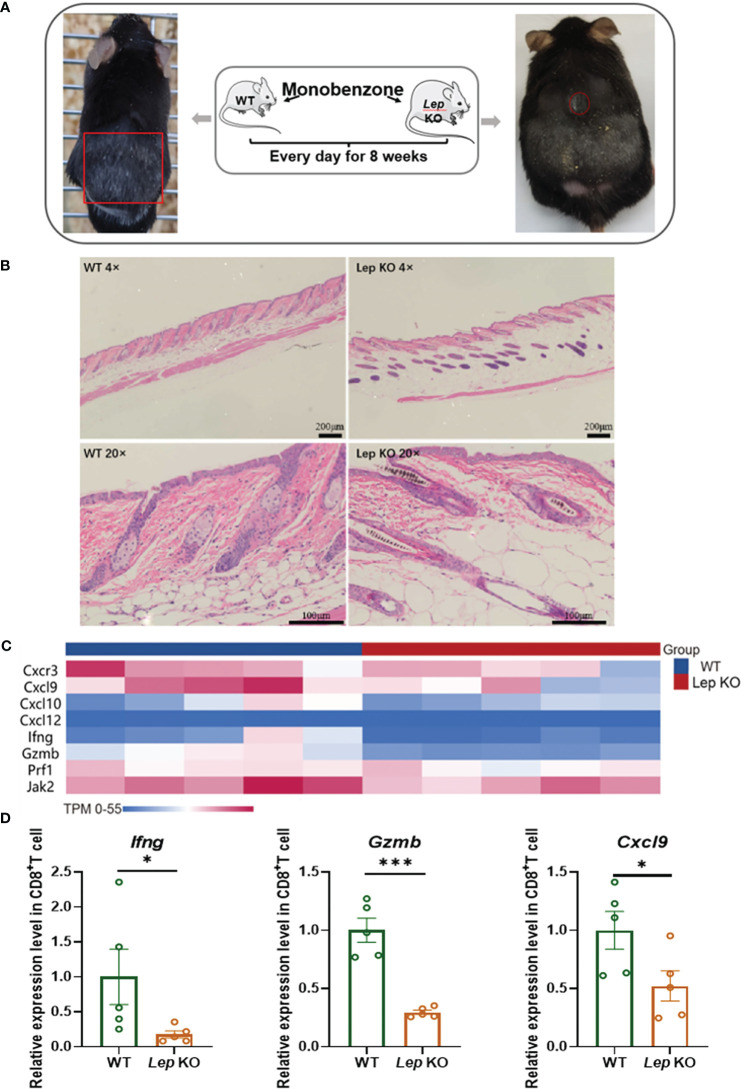
*Leptin* deficiency in CD8^+^ T cells ameliorated vitiligo development in mice. **(A)** The hair decolorization areas of mice with vitiligo induced by monobenzone are shown, marked with red circles. **(B)** HE staining showed that the inflammatory cells of the *Lep* KO mice were significantly reduced compared with the WT group. **(C)** The heatmap shows the differential expression of vitiligo-related genes between WT and *Lep* KO mice after monobenzone was administrated. **(D)** RT-qPCR was used to verify the differentially expressed genes in CD8^+^ T cells from spleens of vitiligo mice induced by monobenzone in the *Lep* KO group and WT group. ^*^P < 0.05, ^***^P < 0.001.

The mouse model of vitiligo was established by monobenzone in C57 BL/6 *Lep* KO (N = 5) mice. Compared with C57 BL/6 WT (N = 5) vitiligo mice, the clinical symptoms of hair depigmentation and inflammatory infiltration of skin lesions in C57 BL/6 *Lep* KO mice were significantly less severe (**Figures 4A, B**). Transcriptome sequencing of CD8^+^ T cells in the mouse model showed that differentially expressed genes were enriched in metabolic and immune pathways (**Figure S1**). The vitiligo-related genes expressed differently between groups and had obvious clustering. Notably, *Ifng*, *Cxcl9* and *Gzmb* had significant expression changes (**Figure 4C**). RNA from CD8^+^ T cells was evaluated by RT-qPCR and *Cxcl9* (p = 0.0497), *Gzmb* (p< 0.001), and *Ifng* (p = 0.0159) were significantly lower with Leptin deficiency (**Figure 4D**). Flow cytometry further demonstrated that *Lep* depletion decreased the expression level of cytotoxic cytokines in CD8^+^ T cells in the vitiligo mouse model, but the results weren’t statistically significant (**Table S10**). In addition, total RNA was extracted from the mice’s skin. RT-qPCR showed that the expression of vitiligo-related genes *Mx1* in C57 BL/6 *Lep* KO mice was significantly lower than that in C57 BL/6 WT mice (p < 0.001, **Figure S2**). In total, *Lep* deficient mice showed reduced clinical symptoms of vitiligo after monobenzone induction and the cytotoxic function of CD8^+^ T cells was weakened.

## 4 Discussion

This study has found a close relationship between vitiligo and lipid metabolism, especially the PPAR signaling pathway. As a key regulator of lipid metabolism, PPAR guides the activation, differentiation, and expansion of various immune cell types (32, 33), and plays an important role in T cell response and the occurrence and development of T cell-mediated autoimmune diseases. PPARα was found to inhibit IFN-γ production by recruiting nuclear receptor corepressor 1 (*NCOR1*) to specific cis-regulatory elements of IFN-γ and reducing histone acetylation at these sites. Furthermore, a novel PPARα antagonist, IS001, was found to increase IFN-γ secretion from NK cells, CD4^+^, and CD8^+^ T cells, and to increase survival in Listeria-infected male mice (34). In this study, the mRNA expression levels of key genes in the PPAR signaling pathway were verified in skin tissues of patients with vitiligo and normal controls. However, RT-qPCR results showed that the mRNA expression levels of most of the key genes in the PPAR signaling pathway in skin tissues of patients with vitiligo were not significantly different from those of normal controls. It is worth noting that the mRNA expression level of *LEPR* was significantly up-regulated in vitiligo skin lesions (P = 0.013), while *LEP* wasn’t. Leptin, as a bridge between lipid metabolism and immune regulation, may play an important role in the development of vitiligo.

This study further explored the specific mechanism by which *LEP* affects CD8^+^ T cells in vitiligo. As a free molecule, the expression of *LEP* in skin is difficult to be clearly shown in immunofluorescence staining. A more effective experimental technique is needed to further explore the localization of *LEP* in skin tissue. As an alternative, *LEPR* was localized by immunofluorescence staining. CD8^+^ T cells were confirmed to express *LEPR* by double staining of *LEPR* and *CD8*. *LEPR* in skin tissues of patients with vitiligo was significantly higher than that of healthy controls (p < 0.05).

In addition, we tried to explore whether Leptin would affect the frequency of CD8^+^ T cell subsets and the secretion of functional cytokines (IFN-γ, perforin, and Granzyme B) by stimulating PBMCs from healthy people with leptin *in vitro*. Cytotoxic cytokines play an important role in the pathogenesis, development and severity of vitiligo. The first is IFN-γ, which is a paracrine inhibitor of melanocytes. Active generalized vitiligo (GV) patients showed increased *IFNG* levels compared to stable GV patients (35). Patients with the early age of onset showed higher *IFNG* expression and female GV patients showed higher *IFNG* and *ICAM1* expression implicating gender biasness and involvement of IFN-γ in early onset of the disease. Vitiligo depigmentation is accompanied by accumulation of autoreactive CD8^+^ T cells in the skin and local IFN-γ production. CD8^+^ T-cell accumulation and depigmentation can be prevented by neutralization of IFN-γ with antibody (36). The IFN-γ-CXCL10-CXCR3 axis may be a new target for developing vitiligo treatments (37). The second is Granzyme B. Downregulation of immunosuppressive gene *GZMB* in the Treg cells of vitiligo patients can lead to the decrease of the inhibitory function of Treg and aggravate the vitiligo (38). Other studies have suggested that perforin and Granzyme B expressed by CD8^+^ T cells can directly lyse target cells, and melanocyte specific cytotoxic CD8^+^ T cells have been detected in blood and skin lesions of vitiligo patients (15, 39). In different vitiligo cells, Granzyme B may play different roles. The RT-qPCR results showed that there was no significant difference in the expression of CD8^+^ T cell-related functional molecules and classical molecules in the pathogenesis of vitiligo. However, the levels of *IFNG*, *PRF1*, *GZMB* and *CXCR3*, *CXCL9*, *CXCL10*, *CXCL12*, and other cytokines also had an upward trend in the vitiligo group. Flow cytometry results showed that Leptin could stimulate CD8^+^ T cells to express more *LEPR*, and *LEPR* showed positive feedback to Leptin. Flow cytometry and ELISA results further confirmed that leptin stimulation induced CD8^+^ T cells to secrete more IFN-γ, perforin, and Granzyme B, thereby enhancing CD8^+^ T cell function.

Previous studies have suggested that leptin is mainly secreted by adipose tissue, so it is positively correlated with BMI. However, previous case-control studies have shown no significant difference in BMI between vitiligo patients and healthy subjects (31). With no significant differences in BMI, we performed an ELISA assay on peripheral blood serum collected from 53 vitiligo patients and 36 healthy subjects. The result showed that the serum leptin concentration of vitiligo patients was significantly lower than that of healthy controls. This result suggested that the secretion of leptin in vitiligo patients was abnormal. In fact, it has been reported that the tumor suppressor protein p53 is overexpressed in both skin and non-skin epidermis of patients with vitiligo (40), and this protein has a potential effect of resisting diet-induced obesity. In addition, patients with vitiligo have higher concentrations of transforming growth factor-β (TGF-β), a molecule known to be a potent repressor of white adipose tissue differentiation (41, 42). The high expression of p53 and TGF-β may partly explain the decrease of serum leptin in patients with vitiligo. Notably, the expression of *LEPR* in the skin tissues of patients with vitiligo was significantly higher than that of healthy controls. We proposed a hypothesis that *Leptin* positively regulates the expression of *LEPR* in vitiligo, and patients with vitiligo have higher uptake and utilization of leptin than healthy controls. This hypothesis needs to be further confirmed in larger skin samples with vitiligo in the future. Serum leptin levels are also associated with *Leptin* Gene Polymorphisms. In a case-control study, SNPs of exons in *LEP* were found to be rare but associated with morbid obesity and altered levels of serum leptin in Kerala, India (43). However, the effect of genetic polymorphism was difficult to quantify in this study. Compared with normal controls, CD8^+^LEPR^+^ T cells in the peripheral blood of patients with vitiligo increased the expression of cytotoxic cytokines. The proportion of IFN-γ, Granzyme B, and perforin in CD8^+^LEPR^+^ T cells was higher than that in CD8^+^LEPR^-^ T cells. The expression of *LEPR* in patients with vitiligo is correlated with the expression of cytotoxic cytokines by CD8^+^ T cells to some extent.

In this study, the vitiligo model was successfully established in both groups of mice. *Lep* KO mice with *Leptin* deficiency had smaller depigmentation areas and longer depigmentation time than C57 BL/6 WT mice, suggesting that *Leptin* plays an important role in the occurrence and development of vitiligo. Similarly, the expression of IFN-γ, perforin, and Granzyme B was lower in *Lep* KO mice with leptin depletion. Interestingly, leptin deficient vitiligo mice also showed significantly different mRNA levels of other vitiligo-related genes compared with control mice, including *Mx1, Prf1, Gzmb*, and Cell adhesion molecule *Cdh1*. Increased IFN-γ secretion in patients can lead to higher intercellular cell adhesion molecule-1 (*ICAM1*) expression (35). Leptin may further promote the occurrence and development of vitiligo disease by affecting cell adhesion molecules.

Notably, abnormal leptin levels do not necessarily cause vitiligo. Leptin alteration has also been found in metabolic diseases or obesity (44), but not everyone with leptin abnormalities develops vitiligo. In fact, vitiligo is caused by multiple factors, including genetic susceptibility, viral infection, melanocyte self-destruction, oxidative stress and melanocyte loss, etc (45). Our research confirmed that abnormal leptin levels can lead to secretion of more cytotoxic factors by CD8^+^ T cells, and leptin deficiency can alleviate the disease symptoms of vitiligo. In conclusion, leptin plays an important role in the pathogenesis and development of vitiligo, but does not constitute sufficient and necessary conditions for the onset of vitiligo.

## 5 Conclusion

In this study, high-throughput transcriptome sequencing was used to screen out the differential metabolic pathways and differentially expressed genes in skin tissues and CD8^+^ T cells of vitiligo and healthy controls. Enrichment analysis verified that lipid metabolism is likely to be closely related to the pathogenesis and disease progression of vitiligo, especially the PPAR signaling pathway, of which *LEP* and *LEPR* are representative genes. We also found that serum leptin was decreased while the expression of *LEPR* was increased in patients with vitiligo. Leptin can enhance the cytotoxic function of CD8^+^ T cells and may promote the occurrence and development of vitiligo diseases by affecting cell adhesion molecules. Due to the limited sample size of this study, the specific mechanism of leptin’s effect on vitiligo by affecting CD8^+^ T cells and other pathways needs to be further studied with a larger sample size.

This study confirmed the effect of leptin on the occurrence and development of vitiligo by enhancing the cytotoxic function of CD8^+^T cells, revealing an inseparable relationship between lipid metabolism pathways and vitiligo. *LEP* or *LEPR* may become a new target for the treatment of vitiligo.

## Data availability statement

The data that support the findings of this study are not publicly available due to their containing information that could compromise the privacy of research participants but are available from the corresponding author.

## Ethics statement

The studies involving human participants were reviewed and approved by the Ethics Committee of The Second Xiangya Hospital of Central South University. Written informed consent to participate in this study was provided by the participants’ legal guardian/next of kin. The animal study was reviewed and approved by the Ethics Committee of The Second Xiangya Hospital of Central South University.

## Author contributions

HZ and HW wrote the protocol and treated the patients. MW, LW, MY, and YC collected data. ZH performed the data analyses. LW drafted the manuscript. All authors contributed to the article and approved the submitted version.
