# Supplementary material for: Leptin deficiency in CD8+ T cells ameliorates non-segmental vitiligo by reducing interferon-γ and Granzyme B
Source: Front Immunol. 2023 May 3;14:1158883. doi: 10.3389/fimmu.2023.1158883 (PMC10191228; doi:10.3389/fimmu.2023.1158883)
Supplement: Supplementary file 4 [file DataSheet_4.docx]

***Supplementary Material***

**Leptin deficiency in CD8^+^ T cells ameliorates vitiligo by reducing IFN-γ and Granzyme B**

**Meiyu Wu^1^†, Lu Wang^1^†, Haijing Wu^1^, Ming Yang^1^, Zhenghao He^1^, Yiran Chen^2^, Huiming Zhang****^1^***

***Correspondence:**

Huiming Zhang, MD,

Department of Dermatology, Hunan Key Laboratory of Medical Epigenomics,

The Second Xiangya Hospital, Central South University,

139 Ren Min Middle Road,

Changsha, Hunan 410011, China

Tel: 8615074951741

E-mail: [huimingzhang@csu.edu.cn](mailto:huimingzhang@csu.edu.cn)

**Supplementary Figures and Tables**

**Table S1:** Basic information of RNA-seq samples

| Group | Number | Gender | Age | BMI |
| --- | --- | --- | --- | --- |
| Vitiligo | Vitiligo1 | female | 23 | 19.56 |
|  | Vitiligo2 | female | 24 | 18.67 |
|  | Vitiligo3 | male | 30 | 20.89 |
| Normal controls | NC1 | female | 21 | 18.49 |
|  | NC2 | female | 28 | 19.13 |
|  | NC3 | female | 33 | 19.78 |

**Table S2:** Basic information of RT-qPCR samples

| Group | Number | Gender | Age | BMI |
| --- | --- | --- | --- | --- |
| Vitiligo | Vitiligo4 | female | 25 | 20.61 |
|  | Vitiligo5 | male | 21 | 22.09 |
|  | Vitiligo6 | male | 22 | 21.38 |
|  | Vitiligo7 | male | 22 | 19.26 |
|  | Vitiligo8 | female | 25 | 19.98 |
|  | Vitiligo9 | female | 20 | 19.29 |
|  | Vitiligo10 | female | 23 | 19.48 |
|  | Vitiligo11 | female | 49 | 18.52 |
|  | Vitiligo12 | male | 16 | 20.05 |
| Normal controls | Vitiligo13 | female | 30 | 20.32 |
|  | Vitiligo14 | female | 32 | 20.31 |
|  | Vitiligo15 | male | 12 | 18.42 |
|  | NC4 | female | 20 | 19.38 |
|  | NC5 | male | 14 | 20.06 |
|  | NC6 | female | 23 | 20.96 |
|  | NC7 | male | 51 | 23.62 |
|  | NC8 | male | 22 | 21.73 |
|  | NC9 | male | 19 | 21.46 |
|  | NC10 | male | 17 | 22.09 |
|  | NC11 | female | 28 | 19.57 |
|  | NC12 | female | 22 | 19.22 |

**Table S3:** Basic information of immunohistochemical samples

| Group | Number | Gender | Age | BMI |
| --- | --- | --- | --- | --- |
| Vitiligo | Vtiligo16 | male | 28 | 20.38 |
|  | Vtiligo17 | female | 51 | 19.43 |
|  | Vtiligo18 | female | 54 | 19.72 |
|  | Vtiligo19 | female | 61 | 19.38 |
|  | Vtiligo20 | male | 14 | 20.28 |
| Normal controls | NC13 | female | 31 | 18.90 |
|  | NC14 | female | 41 | 19.63 |
|  | NC15 | male | 61 | 20.20 |
|  | NC16 | male | 19 | 20.51 |
|  | NC17 | female | 25 | 20.57 |

**Table S4:** Basic information of serum samples for Leptin test

| Health controls | Gender | Age | BMI | Vitiligo | Gender | Age | BMI |
| --- | --- | --- | --- | --- | --- | --- | --- |
| H1 | female | 32 | 22.13 | V-1 | female | 23 | 19.26 |
| H2 | male | 19 | 18.33 | V-2 | male | 26 | 23.18 |
| H3 | female | 37 | 20.15 | V-3 | female | 28 | 22.36 |
| H4 | male | 42 | 22.12 | V-4 | male | 21 | 18.28 |
| H5 | female | 52 | 20.38 | V-5 | female | 54 | 23.07 |
| H6 | female | 21 | 19.78 | V-6 | male | 33 | 20.38 |
| H7 | male | 38 | 22.86 | V-7 | female | 39 | 22.19 |
| H8 | male | 42 | 20.28 | V-8 | male | 36 | 19.06 |
| H9 | female | 42 | 21.26 | V-9 | male | 45 | 22.47 |
| H10 | male | 31 | 22.28 | V-10 | female | 51 | 22.86 |
| H11 | male | 51 | 23.03 | V-11 | female | 53 | 21.36 |
| H12 | female | 44 | 21.22 | V-12 | male | 29 | 22.86 |
| H13 | male | 21 | 18.93 | V-13 | female | 44 | 21.93 |
| H14 | male | 29 | 21.50 | V-14 | male | 38 | 22.64 |
| H15 | female | 26 | 19.14 | V-15 | male | 30 | 19.62 |
| H16 | male | 31 | 22.16 | V-16 | male | 22 | 21.91 |
| H17 | female | 32 | 19.73 | V-17 | female | 19 | 22.58 |
| H18 | female | 38 | 21.47 | V-18 | female | 25 | 21.37 |
| H19 | male | 40 | 22.25 | V-19 | female | 25 | 21.26 |
| H20 | female | 38 | 21.37 | V-20 | female | 56 | 22.03 |
| H21 | male | 38 | 22.84 | V-21 | male | 18 | 23.29 |
| H22 | female | 37 | 23.07 | V-22 | male | 18 | 22.08 |
| H23 | female | 36 | 18.43 | V-23 | male | 47 | 21.36 |
| H24 | female | 39 | 19.72 | V-24 | male | 21 | 21.50 |
| H25 | male | 35 | 20.16 | V-25 | male | 35 | 21.79 |
| H26 | male | 38 | 23.37 | V-26 | male | 16 | 22.72 |
| H27 | male | 38 | 21.45 | V-27 | female | 35 | 20.58 |
| H28 | female | 39 | 20.37 | V-28 | female | 34 | 19.05 |
| H29 | female | 40 | 20.20 | V-29 | male | 27 | 21.95 |
| H30 | male | 40 | 22.78 | V-30 | female | 50 | 20.81 |
| H31 | male | 40 | 23.12 | V-31 | female | 14 | 18.73 |
| H32 | female | 39 | 22.08 | V-32 | male | 44 | 22.22 |
| H33 | female | 39 | 18.98 | V-33 | male | 54 | 24.16 |
| H34 | male | 39 | 21.36 | V-34 | female | 33 | 22.86 |
| H35 | female | 39 | 23.05 | V-35 | male | 23 | 21.39 |
| H36 | female | 41 | 22.38 | V-36 | female | 22 | 20.32 |
|  |  |  |  | V-37 | female | 24 | 20.70 |
|  |  |  |  | V-38 | male | 37 | 22.58 |
|  |  |  |  | V-39 | female | 35 | 23.31 |
|  |  |  |  | V-40 | female | 25 | 19.15 |
|  |  |  |  | V-41 | male | 28 | 21.83 |
|  |  |  |  | V-42 | female | 22 | 19.29 |
|  |  |  |  | V-43 | male | 49 | 20.30 |
|  |  |  |  | V-44 | female | 29 | 20.06 |
|  |  |  |  | V-45 | female | 20 | 20.31 |
|  |  |  |  | V-46 | female | 41 | 21.36 |
|  |  |  |  | V-47 | male | 18 | 21.22 |
|  |  |  |  | V-48 | male | 45 | 22.72 |
|  |  |  |  | V-49 | female | 24 | 18.87 |
|  |  |  |  | V-50 | female | 35 | 21.37 |
|  |  |  |  | V-51 | female | 59 | 21.64 |
|  |  |  |  | V-52 | male | 37 | 21.46 |
|  |  |  |  | V-53 | female | 53 | 22.06 |

**Table S5:** Basic information of peripheral blood samples

| Number | Gender | Age | BMI | Volume |
| --- | --- | --- | --- | --- |
| 1 | female | 24 | 19.32 | 20 mL |
| 2 | male | 24 | 20.58 | 20 mL |
| 3 | female | 31 | 21.33 | 20 mL |
| 4 | female | 46 | 20.36 | 20 mL |
| 5 | male | 33 | 21.47 | 20 mL |

**Table S6:** Primer sequences for RT-qPCR

| Species | Gene | Sequence（5’→ 3’） |
| --- | --- | --- |
| Human | *GAPDH* | Forward: GCACCGTCAAGGCTGAGAAC |
|  |  | Reverse: TGGTGAAGACGCCAGTCTCTA |
| Human | *CD36* | Forward: CTTTGGCTTAATGAGACTGGGAC |
|  |  | Reverse: GCAACAAACATCACCACACCA |
| Human | *LEP* | Forward: TGCCTTCCAGAAACGTGATCC |
|  |  | Reverse: CTCTGTGGAGTAGCCTGAAGC |
| Human | *LEPR* | Forward: CTCTGTGGAGTAGCCTGAAGC |
|  |  | Reverse: CTCTGTGGAGTAGCCTGAAGC |
| Human | *OAS1* | Forward: TGTCCAAGGTGGTAAAGGGTG |
|  |  | Reverse: TGTCCAAGGTGGTAAAGGGTG |
| Human | *CPT2* | Forward: CATACAAGCTACATTTCGGGACC |
|  |  | Reverse: AGCCCGGAGTGTCTTCAGAA |
| Human | *DAGT2* | Forward: ATTGCTGGCTCATCGCTGT |
|  |  | Reverse: GGGAAAGTAGTCTCGAAAGTAGC |
| Human | *SLC25A1* | Forward: ACGGGGTTAGGGAGATTGTG |
|  |  | Reverse: GCCTGCAATAGCTCCGAAGA |
| Human | *STAT3* | Forward: CCCAGGTAAAAGGCCCAGG |
|  |  | Reverse: GTGTTCCCACTGATCCCAGAT |
| Human | *PPARG* | Forward: GGGATCAGCTCCGTGGATCT |
|  |  | Reverse: TGCACTTTGGTACTCTTGAAGTT |
| Human | *ACOT4* | Forward: CCCAGGTAAAAGGCCCAGG |
|  |  | Reverse: GTGTTCCCACTGATCCCAGAT |
| Human | *ANGPTL4* | Forward: GGCTCAGTGGACTTCAACCG |
|  |  | Reverse: CCGTGATGCTATGCACCTTCT |
| Human | *SLC25A20* | Forward: GACACGGTCAAGGTCCGAC |
|  |  | Reverse: GCAGCCATTCCCCGATATAGC |
| Human | *SCD* | Forward: GCCCCTCTACTTGGAAGACGA |
|  |  | Reverse: AAGTGATCCCATACAGGGCTC |
| Human | *ACADM* | Forward: GGAAGCAGATACCCCAGGAAT |
|  |  | Reverse: AGCTCCGTCACCAATTAAAACAT |
| Human | *ACOT2* | Forward: CGTCCCGGCTGTACCAATG |
|  |  | Reverse: GGAACCCTAATGATCTGACCAAC |
| Human | *ACSS* | Forward: CCGGTCGTGACCTTGATTGG |
|  |  | Reverse: CGTTGTGCCAGATGTGTAAAGA |
| Human | *ACSL1* | Forward: CTTATGGGCTTCGGAGCTTTT |
|  |  | Reverse: CAAGTAGTGCGGATCTTCGTG |
| Human | IFNG | Forward: CAGATGTAGCGGATAATGGA |
|  |  | Reverse: TCACTTGGATGAGTTCATGT |
| Human | Granzyme B | Forward: TACCATTGAGTTGTGCGTGGG |
|  |  | Reverse: GCCATTGTTTCGTCCATAGGAGA |
| Human | PRF1 | Forward: GACTGCCTGACTGTCGAGG |
|  |  | Reverse: TCCCGGTAGGTTTGGTGGAA |
| Human | CXCR3 | Forward: CCACCTAGCTGTAGCAGACAC |
|  |  | Reverse: AGGGCTCCTGCGTAGAAGTT |
| Human | CXCL9 | Forward: CCAGTAGTGAGAAAGGGTCGC |
|  |  | Reverse: AGGGCTTGGGGCAAATTGTT |
| Human | CXCL10 | Forward: GTGGCATTCAAGGAGTACCTC |
|  |  | Reverse: TGATGGCCTTCGATTCTGGATT |
| Human | CXCL12 | Forward: ATTCTCAACACTCCAAACTGTGC |
|  |  | Reverse: ACTTTAGCTTCGGGTCAATGC |
| House mouse | Gapdh | Foward: AGGTCGGTGTGAACGGATTTG |
|  |  | Reverse: TGTAGACCATGTAGTTGAGGTCA |
| House mouse | *Ifng* | Foward: ATGAACGCTACACACTGCATC |
|  |  | Reverse: CCATCCTTTTGCCAGTTCCTC |
| House mouse | *Gzmb* | Foward: CCACTCTCGACCCTACATGG |
|  |  | Reverse: GGCCCCCAAAGTGACATTTATT |
| House mouse | *Prf1* | Foward: AGCACAAGTTCGTGCCAGG |
|  |  | Reverse: GCGTCTCTCATTAGGGAGTTTTT |
| House mouse | *Cxcr3* | Foward: TACCTTGAGGTTAGTGAACGTCA |
|  |  | Reverse: CGCTCTCGTTTTCCCCATAATC |
| House mouse | *Cxcl9* | Foward: TCCTTTTGGGCATCATCTTCC |
|  |  | Reverse: TTTGTAGTGGATCGTGCCTCG |
| House mouse | *Cxcl10* | Foward: CCAAGTGCTGCCGTCATTTTC |
|  |  | Foward: GGCTCGCAGGGATGATTTCAA |
| House mouse | *Cxcl12* | Foward: TGCATCAGTGACGGTAAACCA |
|  |  | Reverse: TTCTTCAGCCGTGCAACAATC |
| House mouse | *Mif* | Foward: GCCAGAGGGGTTTCTGTCG |
|  |  | Reverse: GTTCGTGCCGCTAAAAGTCA |
| House mouse | *Mx1* | Foward: GACCATAGGGGTCTTGACCAA |
|  |  | Reverse: AGACTTGCTCTTTCTGAAAAGCC |
| House mouse | *Icam5* | Foward: TCCGAACTTTCCAGCGACC |
|  |  | Reverse: CTACGAAACTGCGGCGAATC |
| House mouse | *Cdh1* | Foward: CAGGTCTCCTCATGGCTTTGC |
|  |  | Reverse: CTTCCGAAAAGAAGGCTGTCC |
| House mouse | *Hsp70* | Foward: GAGATCGACTCTCTGTTCGAGG |
|  |  | Reverse: GCCCGTTGAAGAAGTCCTG |
| House mouse | *Il15* | Foward: ACATCCATCTCGTGCTACTTGT |
|  |  | Reverse: GCCTCTGTTTTAGGGAGACCT |

**Table S7:** Comparison of gene expression in skin lesions between vitiligo patients and normal controls by RT-qPCR

| Group | Gene Name | Mean ± SEM | P Value |
| --- | --- | --- | --- |
| NC | *LEP* | 1.00 ± 0.39 | *P* > 0.05 |
| Vitiligo |  | 4.37 ± 1.94 |  |
| NC | *LEPR* | 0.80 ± 0.18 | *P* < 0.05 |
| Vitiligo |  | 1.79 ± 0.28 |  |
| NC | *CD36* | 0.30 ± 0.14 | *P* > 0.05 |
| Vitiligo |  | 0.45 ± 0.11 |  |
| NC | *OAS1* | 6.55 ± 0.60 | *P* > 0.05 |
| Vitiligo |  | 6.08 ± 0.72 |  |
| NC | *CPT2* | 2.11 ± 0.51 | *P* > 0.05 |
| Vitiligo |  | 2.56 ± 1.24 |  |
| NC | *DAGT2* | 8.96 ± 3.88 | *P* > 0.05 |
| Vitiligo |  | 11.34 ± 4.13 |  |
| NC | *SLC25A1* | 1.03 ± 0.51 | *P* > 0.05 |
| Vitiligo |  | 1.17 ± 0.48 |  |
| NC | *STAT3* | 11.29 ± 2.50 | *P* > 0.05 |
| Vitiligo |  | 12.51 ± 1.39 |  |
| NC | *PPARG* | 0.17 ± 0.04 | *P* > 0.05 |
| Vitiligo |  | 0.25 ± 0.03 |  |
| NC | *ACOT4* | 0.54 ± 0.12 | *P* > 0.05 |
| Vitiligo |  | 0.56 ± 0.16 |  |
| NC | *ANGPTL4* | 1.92 ± 1.78 | *P* > 0.05 |
| Vitiligo |  | 2.26 ± 1.87 |  |
| NC | *SLC25A20* | 1.30 ± 0.96 | *P* > 0.05 |
| Vitiligo |  | 2.40 ± 1.79 |  |
| NC | *SCD* | 0.39 ± 0.30 | *P* > 0.05 |
| Vitiligo |  | 0.90 ± 0.28 |  |
| NC | *ACADM* | 1.05 ± 0.47 | *P* > 0.05 |
| Vitiligo |  | 3.17 ± 0.83 |  |
| NC | *ACOT2* | 0.003 ± 0.001 | *P* < 0.05 |
| Vitiligo |  | 0.006 ± 0.001 |  |
| NC | *CPT2* | 0.31 ± 0.25 | *P* > 0.05 |
| Vitiligo |  | 0.11 ± 0.05 |  |
| NC | *ACSS* | 3.63 ± 2.01 | *P* > 0.05 |
| Vitiligo |  | 8.75 ± 2.60 |  |
| NC | *ACSL1* | 26.46 ± 9.14 | *P* > 0.05 |
| Vitiligo |  | 23.86 ± 6.77 |  |

**Table S8:** Comparison of CD8^+^ T cell subsets frequency in PBMCs from vitiligo patients and healthy controls by flow cytometry

| Group | T cell subset | Mean ± SEM | P Value |
| --- | --- | --- | --- |
| Vitiligo | CD8^+^ | 37.38 ± 5.068 | *P* > 0.05 |
| HC | CD8^+^ | 35.66 ± 3.084 |  |
| Vitiligo | CD8^+^LEPR^+^ | 27.19 ± 13.43 | *P* > 0.05 |
| HC | CD8^+^LEPR^+^ | 12.13 ± 3.046 |  |
| Vitiligo | CD8^+^ LEPR^+^Perforin^+^ | 14.12 ± 5.494 | *P* > 0.05 |
| HC | CD8^+^ LEPR^+^Perforin^+^ | 7.528 ± 1.912 |  |
| Vitiligo | CD8^+^ LEPR^+^Granzyme B ^+^ | 69.12 ± 9.412 | *P* > 0.05 |
| HC | CD8^+^ LEPR^+^Granzyme B ^+^ | 71.18 ± 3.159 |  |
| Vitiligo | CD8^+^ LEPR^+^IFN-γ^+^ | 9.196 ± 1.372 | *P* < 0.05 |
| HC | CD8^+^ LEPR^+^IFN-γ^+^ | 4.102 ± 1.066 |  |
| Vitiligo | CD8^+^ LEPR^-^perforin^+^ | 6.750 ± 2.443 | *P* > 0.05 |
| HC | CD8^+^ LEPR^-^ perforin^+^ | 2.056 ± 0.7635 |  |
| Vitiligo | CD8^+^ LEPR^-^ Granzyme B ^+^ | 40.06 ± 16.31 | *P* > 0.05 |
| HC | CD8^+^ LEPR^-^ Granzyme B ^+^ | 41.16 ± 5.150 |  |
| Vitiligo | CD8^+^ LEPR^-^IFN-γ^+^ | 5.388 ± 0.8854 | *P* > 0.05 |
| HC | CD8^+^ LEPR^-^IFN-γ^+^ | 5.528 ± 1.371 |  |
| Vitiligo | CD8^+^LEPR^+^Perforin^+^ | 14.12 ± 5.494 | *P* > 0.05 |
| Vitiligo | CD8^+^LEPR^-^Perforin^+^ | 6.750 ± 2.443 |  |
| Vitiligo | CD8^+^LEPR^+^Granzyme B^+^ | 69.12 ± 9.412 | *P* > 0.05 |
| Vitiligo | CD8^+^LEPR^-^ Granzyme B ^+^ | 40.06 ± 16.31 |  |
| Vitiligo | CD8^+^LEPR^+^ IFN-γ^+^ | 9.196 ± 1.372 | *P* < 0.05 |
| Vitiligo | CD8^+^LEPR^-^ IFN-γ^+^ | 5.388 ± 0.8854 |  |

**Table S9:** Frequency of CD8^+^ T cell subsets after Leptin stimulation in normal PBMCs by flow cytometry

| Group | T cell subset | Mean ± SEM | P Value |
| --- | --- | --- | --- |
| PHA + Leptin | CD8^+^ | 39.32 ± 2.559 | *P* < 0.01 |
| PHA | CD8^+^ | 30.38 ± 0.9410 |  |
| PHA + Leptin | CD8^+^LEPR^+^ | 26.24 ± 3.481 | *P* < 0.05 |
| PHA | CD8^+^LEPR^+^ | 19.32 ± 2.014 |  |
| PHA + Leptin | CD8^+^LEPR^+^perforin^+^ | 28.62 ± 1.562 | *P* < 0.01 |
| PHA | CD8^+^LEPR^+^perforin^+^ | 20.88 ± 2.435 |  |
| PHA + Leptin | CD8^+^LEPR^+^granzyme B^+^ | 42.92 ± 2.158 | *P* > 0.05 |
| PHA | CD8^+^ LEPR^+^granzyme B^+^ | 44.30 ± 2.029 |  |
| PHA + Leptin | CD8^+^ LEPR^+^IFN-γ^+^ | 3.154 ± 0.6288 | *P* > 0.05 |
| PHA | CD8^+^ LEPR^+^IFN-γ^+^ | 1.712 ± 0.2256 |  |
| PHA + Leptin | CD8^+^LEPR^-^perforin^+^ | 66.040 ± 0.9955 | *P* > 0.05 |
| PHA | CD8^+^LEPR^-^perforin^+^ | .372 ± 1.115 |  |
| PHA + Leptin | CD8^+^LEPR^-^granzyme B^+^ | 17.19 ± 2.883 | *P* > 0.05 |
| PHA | CD8^+^ LEPR^-^granzyme B^+^ | 15.86 ± 1.912 |  |
| PHA + Leptin | CD8^+^ LEPR^-^IFN-γ^+^ | 2.268 ± 0.3608 | *P* > 0.05 |
| PHA | CD8^+^ LEPR‑IFN-γ^+^ | 1.938 ± 0.2104 |  |
| PHA + Leptin | CD8^+^ LEPR^+^granzyme B^+^ | 45.54 ± 1.482 | *P* < 0.001 |
| PHA + Leptin | CD8^+^ LEPR^-^granzyme B^+^ | 17.19 ± 2.883 |  |
| PHA + Leptin | CD8^+^LEPR^+^perforin^+^ | 28.62 ± 1.562 | *P* < 0.001 |
| PHA + Leptin | CD8^+^LEPR^-^perforin^+^ | 6.040 ± 0.9955 |  |
| PHA + Leptin | CD8^+^ LEPR^+^IFN-γ^+^ | 3.154 ± 0.6288 | *P* > 0.05 |
| PHA + Leptin | CD8^+^ LEPR-IFN-γ^+^ | 2.268 ± 0.3608 |  |

**Table S10:** Frequency of splenic T cell subsets from *Lep* KO and WT mice

| Group | T cell subset | Mean ± SEM | P Value |
| --- | --- | --- | --- |
| *Lep* KO | CD8^+^ | 17.66 ± 2.594 | *P* > 0.05 |
| WT | CD8^+^ | 17.18 ± 1.726 |  |
| *Lep* KO | CD8^+^perforin^+^ | 0.4040 ± 0.1633 | *P* > 0.05 |
| WT | CD8^+^perforin^+^ | 0.5000 ± 0.1190 |  |
| *Lep* KO | CD8^+^Granzyme B^+^ | 0.4532 ± 0.3113 | *P* > 0.05 |
| WT | CD8^+^Granzyme B^+^ | 1.072 ± 0.3849 |  |
| *Lep* KO | CD8^+^IFN-γ^+^ | 1.548 ± 0.3591 | *P* > 0.05 |
| WT | CD8^+^IFN-γ^+^ | 1.800 ± 0.3573 |  |


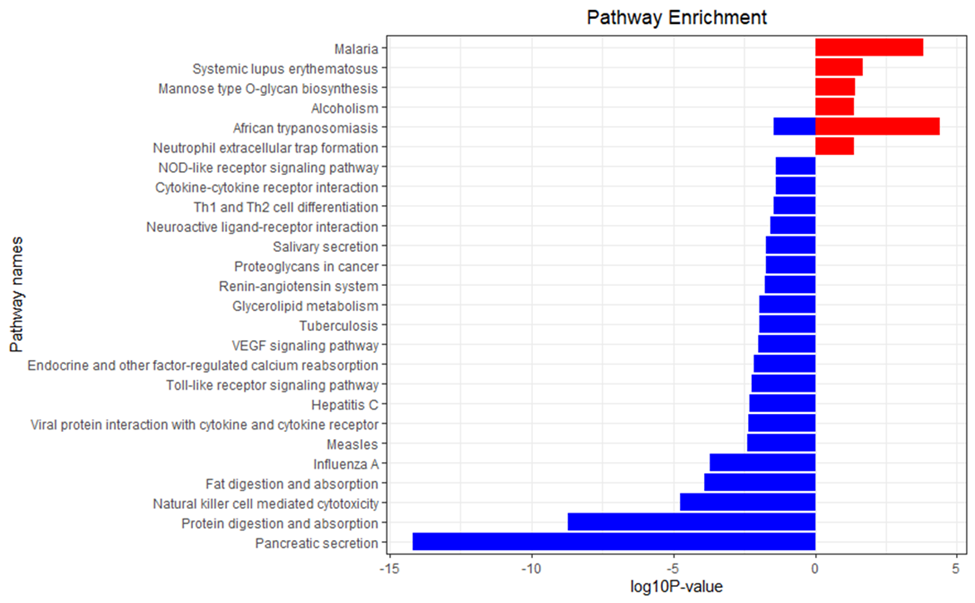


**Figure S1:** The results of the pathway enrichment analysis are shown. The vertical axis shows the names of different signaling pathways. The red bar indicates the high expression while the blue bar indicates the low expression.


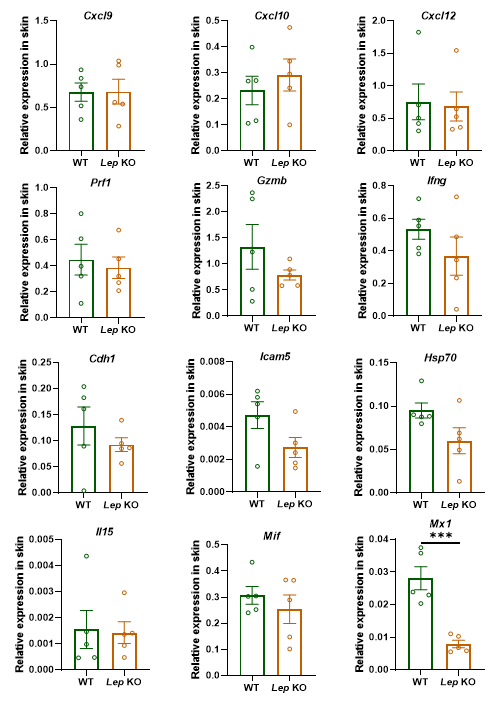


**Figure S2:** RT-qPCR results of total RNA extracted from the skin of C57 BL/6 Lep KO (N=5) and C57 BL/6 WT mice (N=5) are shown.
